# Racial Differences in Hepatocellular Carcinoma Incidence and Risk Factors among a Low Socioeconomic Population

**DOI:** 10.3390/cancers13153710

**Published:** 2021-07-23

**Authors:** Sylvie Muhimpundu, Rebecca Baqiyyah N. Conway, Shaneda Warren Andersen, Loren Lipworth, Mark D. Steinwandel, William J. Blot, Xiao-Ou Shu, Staci L. Sudenga

**Affiliations:** 1 Division of Epidemiology, Vanderbilt University Medical Center, Nashville, TN 37232, USA; sylvie.muhimpundu@vanderbilt.edu (S.M.); loren.lipworth@vumc.org (L.L.); william.j.blot@vumc.org (W.J.B.); xiao-ou.shu@vumc.org (X.-O.S.); 2 School of Community and Rural Health, University of Texas Health Science Center at Tyler, Tyler, TX 75708, USA; rebecca.conway@aaepi.org; 3 American Academy of Epidemiology, Inc., Tyler, TX 75701, USA; 4 Department of Population Health Sciences, School of Medicine and Public Health, University of Wisconsin-Madison, Madison, WI 53706, USA; snandersen@wisc.edu; 5 Cancer Prevention and Control, University of Wisconsin Carbone Cancer Center, Madison, WI 53706, USA; 6 International Epidemiology Institute, Rockville, MD 20850, USA; mark.d.steinwandel@vumc.org

**Keywords:** cancer risk, southeast, racial disparity, SCCS

## Abstract

**Simple Summary:**

Liver cancer incidence in the United States is higher among African Americans compared to White Americans. The determinants of racial disparities in liver cancer incidence are not clear. Using data from White and African Americans from low socioeconomic backgrounds, we compared the prevalence of known liver cancer risk factors by race and assessed factors associated with liver cancer incidence. Understanding liver cancer risk differences can assist prevention strategies that target people at high risk, potentially based on risk factors that differ by race.

**Abstract:**

The purpose of this study was to examine differences in risk factors associated with hepatocellular carcinoma (HCC) among White and African Americans from low socioeconomic backgrounds in the Southern Community Cohort Study (SCCS). The SCCS is a prospective cohort study with participants from the southeastern US. HCC incidence rates were calculated. Multivariable Cox regression was used to calculate HCC-adjusted hazard ratios (aHR) associated with known baseline HCC risk factors for White and African Americans, separately. There were 294 incident HCC. The incidence rate ratio for HCC was higher (IRR = 1.4, 95%CI: 1.1–1.9) in African Americans compared to White Americans. White Americans saw a stronger association between self-reported hepatitis C virus (aHR = 19.24, 95%CI: 10.58–35.00) and diabetes (aHR = 3.55, 95%CI: 1.96–6.43) for the development of HCC compared to African Americans (aHR = 7.73, 95%CI: 5.71–10.47 and aHR = 1.48, 95%CI: 1.06–2.06, respectively) even though the prevalence of these risk factors was similar between races. Smoking (aHR = 2.91, 95%CI: 1.87–4.52) and heavy alcohol consumption (aHR = 1.59, 95%CI: 1.19–2.11) were significantly associated with HCC risk among African Americans only. In this large prospective cohort, we observed racial differences in HCC incidence and risk factors associated with HCC among White and African Americans.

## 1. Introduction

The incidence of liver cancer has been shown to be increasing over the last decade in many parts of the world including the United States (US) [1,2,3]. Liver cancer has a 5-year relative cancer survival rate at 18%, making it the fifth leading cause of cancer death in men and seventh in women in the US in 2016 [4,5]. By 2030, liver cancer is projected to be the third leading cause of cancer-related death in the US [4]. This increasing liver cancer incidence and mortality is likely due to the high prevalence of hepatitis C virus (HCV) infections in the “baby boomer” generation (born from 1945 to 1965) [6,7,8] and the increasing incidence of non-alcoholic fatty liver disease [9].

There are differences in liver cancer incidence and mortality rates by race and ethnicity in the US. Liver cancer incidence rates are higher among African Americans (10.2 per 100,000) compared to White Americans (6.3 per 100,000) [8]. Although liver cancer stage at diagnosis is similar amongst White and African Americans, the 5-year survival in African Americans (21%) was significantly lower for all stages combined compared to White Americans (25%) [8,10]. The determinants of racial disparities in liver cancer incidence are not clear. Observed differences in liver cancer trends may reflect race/ethnicity-specific differences in the prevalence of the risk factors for liver cancer. Known liver cancer risk factors include chronic HCV, chronic hepatitis B virus (HBV), non-alcoholic steatohepatitis, alcoholic liver disease, diabetes, and obesity [8,10,11,12,13,14,15]. Hepatocellular carcinoma (HCC) accounts for 75–85% of liver cancers [2]. The purpose of this analysis is to examine racial differences in self-reported risk factors (e.g., smoking, alcohol, hepatitis infection, diabetes, and obesity) for HCC incidence among White and African Americans participating in the Southern Community Cohort Study (SCCS), a cohort of individuals with a low socioeconomic background.

## 2. Materials and Methods

### 2.1. Study Population

**Figure 1:**
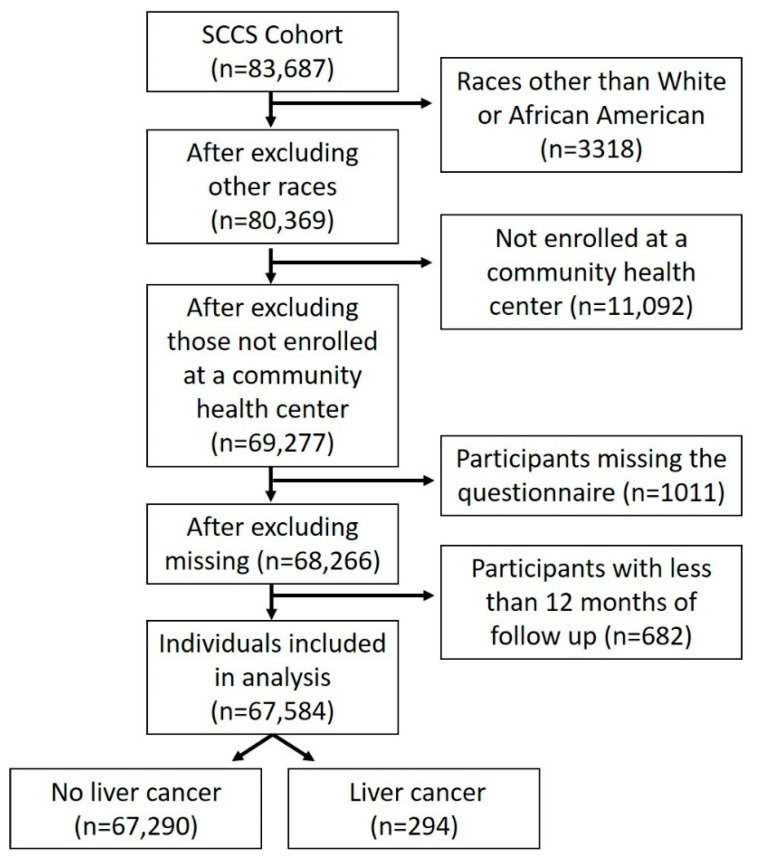
Flow chart of study participants. Exclusion and inclusion criteria are shown.

The SCCS is a prospective study designed to assess the determinants of racial and socioeconomic differences in cancer and other diseases [16,17]. Participants aged 40 to 79 years were recruited across 12 states: Alabama, Arkansas, Florida, Georgia, Kentucky, Louisiana, Mississippi, North Carolina, South Carolina, Tennessee, Virginia, and West Virginia. Cohort recruitment took place between 2002 and 2009. Most participants (~85%) were recruited from community health centers (CHCs) in the 12 states and completed a comprehensive baseline interview. The remainder of participants were recruited using stratified random samples of the residents in the 12 states and completed an identical mailed questionnaire. For this analysis, we chose to only include participants recruited from CHC to focus on persons that were underinsured or uninsured (Figure 1). The baseline survey collected self-reported information on disease determinants such as the participant’s demographic factors, history of prior medical conditions, diet, and lifestyle. To be eligible, participants must have been English-speaking, and not under treatment for cancer within the past year (with the exception of non-melanoma skin cancer). The institutional review boards at Vanderbilt University Medical Center and Meharry Medical College approved the study protocol.

### 2.2. Hepatocellular Carcinoma (HCC) Incidence

State Cancer Registries and the National Death Index (NDI) were the primary means of ascertainment of incident HCC diagnoses [16]. SCCS data can be linked with the 12 state cancer registries from which the participants were recruited. It is possible that participants can move out of those twelve states, so we also connected with NDI to identify those additional cases. Liver cancers were identified using the International Classification of Diseases (ICD) 10 code of C22.0 for hepatocellular carcinoma. The participants without HCC were linked to the Social Security Administration (SSA) for determination of current vital status. Cohort member information for those known through SSA to have died (or whose current status is unknown to SSA) was sent to the NDI to ascertain cause (or fact and cause) of death [17]. Cohort data were updated until 31 December 2017.

For participants with an incident HCC diagnosis, follow-up time was calculated from date of cohort enrollment to date of HCC diagnosis for those identified in the cancer registry (*n* = 270) or date of death from HCC for those identified in the NDI (*n* = 24). Follow-up time for participants without an incident HCC diagnosis was calculated from date of cohort enrollment to the date of death or date of vital status.

### 2.3. Population for Analysis

For the current study, we excluded participants with a prior history of liver cancer and those that were missing race and ethnicity (Figure 1). We also excluded participants not from Non-Hispanic White and Non-Hispanic African American ethnic groups (*n* = 3318), participants who were not enrolled at the community health centers (*n* = 11,092), participants that did not have the baseline questionnaire (*n* = 1011), or with a follow-up period of fewer than 12 months (*n* = 682). After all exclusions, 67,584 participants (72% Non-Hispanic African Americans and 28% Non-Hispanic Whites) were included in the analyses. We will refer to Non-Hispanic Whites as White Americans and Non-Hispanic African Americans as African Americans for brevity. Participants self-identified as being African American or White. These self-identified racial categories were not based on genetic characterization. Baseline demographic information that was self-reported in the baseline survey was used to determine risk factors associated with HCC risk.

### 2.4. Statistical Analysis

We used baseline questionnaire responses for our exposures of interest. In this analysis, body mass index (BMI) was calculated as weight in kilograms (kg) over height (m^2^) and categorized as normal (<25 kg/m^2^), overweight (25 to 30 kg/m^2^), and obese (≥30 kg/m^2^). Self-reported age at baseline was analyzed using restricted cubic splines with 3 knots (White American model) or 4 knots (African American model). Participants were asked how often they drank alcohol in the past year and how many drinks they typically had in a day when drinking. We categorized heavy alcohol drinkers as women with on average more than one alcoholic drink per day and men with on average more than two alcoholic drinks per day compared to women and men drinking less than that per day [18]. We analyzed alcohol both as a continuous variable and categorical variable and found similar results between the classifications. We report the analyses using a categorical variable for alcohol in this manuscript. Current cigarette smoking status was classified as current, former, and never in our analyses. We assessed cigarette smoking intensity (pack-years) and found similar results to the cigarette smoking status. Hepatitis B virus (HBV), HCV, and diabetes were all based on self-report with the participants being asked “Has a doctor ever told you that you have this virus/disease?”. We did not ask if the participant had ever been tested for HBV, HCV, or diabetes.

The age-adjusted incidence rate was calculated as the number of HCC incident cases divided by the follow-up years, and 95% confidence intervals (CIs) were estimated. Incidence rate ratios (IRR) and 95%CIs were also calculated comparing incidence rates between White and African Americans. Cox proportional hazards regression was used to compute hazard ratios (HRs) and 95%CIs for the incidence of HCC stratified by race. Known HCC risk factors and potential confounders (age, gender, BMI, education, household income, HBV, HCV, smoking, alcohol, employment status, and diabetes) were identified a priori and assessed within the models. We assessed the interaction between several known HCC risk factors, specifically between diabetes and HCV, and between smoking and alcohol. We also assessed the interaction between the known HCC risk factors and race. The proportionality assumption model using Schoenfeld residuals was assessed and held for the White American model but did not hold for the African American model. When the African American model was stratified by gender, the proportionality assumption was satisfied. Separate unadjusted and adjusted models for African Americans by gender are provided as supplemental tables. All the data analysis was conducted using STATA/IC 15.0.

## 3. Results

### 3.1. Study Population

Overall, there were 294 incident HCC among White (*n* = 57) and African Americans (*n* = 237). Baseline characteristics of the study population by race are shown in Table 1. White and African Americans had similar prevalence of obese participants; 45% compared to 46%, respectively. Household income <USD 15,000 was more prominent in the African American population (62%) compared to the White Americans (56%). Self-reported hepatitis C and hepatitis B infection prevalence was reported more often by White Americans than African Americans. Diabetes prevalence was also similar between White (21%) and African Americans (22%). The prevalence of currently smoking was similar between African Americans (44%) and White Americans (43%), but African Americans had a larger proportion that never smoked (36%) compared to White Americans (31%). African Americans had a higher prevalence of heavy alcohol drinkers per day (20%) compared to White Americans (11%). Being currently employed was higher among African Americans (38%) compared to White Americans (33%). We found that ~80% of both White and African American 65 and older were not currently employed.

### 3.2. Incidence Rates

The overall age-adjusted incidence rate of HCC was 45.5 per 100,000 person-years (95%CI: 40.3–50.7) among our participants aged 40–79 living in the southeastern United States. African Americans had higher age-adjusted incidence rates of HCC (IR = 49.8 per 100,000 person-years) compared to White Americans (IR = 33.5 per 100,000 person-years) with an age-adjusted incidence rate ratio (IRR) of 1.48 (95%CI 1.1–2.0).

### 3.3. Cox Regression Model

Table 2 and Table 3 show the multivariable-adjusted hazard ratios of HCC by race. After adjusting for potential risk factors and confounders with age included using restricted cubic splines, males were more likely to develop HCC than females in both African Americans (adjusted hazard ratio (aHR) = 2.05, 95%CI: 1.50–2.80) and White Americans (aHR = 2.83, 95%CI: 1.58–5.07). HCV was significantly associated with HCC in both racial groups, with a more pronounced association among White Americans (aHR = 19.24, 95%CI: 10.58–35.00) than African Americans (aHR = 7.73, 95%CI: 5.71–10.47). In a combined race model assessing race and HCV and HCC, the interaction between race and HCV was not significant (*p* = 0.08). Similarly, diabetes was significantly associated with HCC in both racial groups, but with a stronger association among White Americans (aHR = 3.55, 95%CI: 1.96–6.43) than African Americans (aHR = 1.48, 95%CI: 1.06–2.06). The interaction between race and diabetes in a combined race model assessing race and diabetes and HCC was significant (*p* = 0.01). Among African Americans, current cigarette smokers (aHR = 2.91, 95%CI: 1.87–4.52) had an increased risk of HCC compared to never smokers, but smoking was not significantly associated with HCC risk among White Americans. In a combined race model assessing race and smoking and HCC, the interaction between current smokers and race was significant (*p* = 0.04). Furthermore, heavy alcohol consumption was significantly associated with HCC risk in African Americans only (aHR = 1.59, 95%CI: 1.19–2.11). BMI was not significantly associated with HCC in White Americans and obese African Americans were at a lower risk for HCC (aHR = 0.65, 95%CI: 0.45–0.95).

## 4. Discussion

In this large ongoing cohort study of mostly low socioeconomic White and African American participants living in the southeastern US, we found racial differences in self-reported risk factors associated with HCC incidence. While the prevalence of several known risk factors for HCC were generally similar between White and African Americans, HCC incidence rates were higher among African Americans compared to White Americans. White Americans saw a greater effect between self-reported HCV and diabetes for the development of HCC compared to African Americans, while smoking and alcohol use were significantly associated with HCC risk among African Americans only.

We found that males had a higher incidence of HCC than females in both White and African Americans. Gender differences in HCC incidence may reflect the higher prevalence of HCC risk factors in males than females (Table S3). An additional possible explanation for the observed gender disparity in HCC incidence is the higher estrogen levels in females compared to males. Studies suggest that estrogen reduces HCC by inhibiting the production of interleukin-6 (IL-6), a multifunctional cytokine that may be causal or contributory to HCC [19,20,21].

Previous studies have shown that diabetes is associated with a two- to three-fold increased risk of HCC and other conditions such as chronic non-alcoholic liver disease [22]. These findings corroborate with our results; diabetes had a positive association with HCC. The association was statistically significant in both White and African Americans but was stronger in White Americans compared to African Americans. Although the prevalence of self-reported diabetes at baseline was higher in African Americans (22.0%, 95%CI 21.6–22.4%) compared to White Americans (20.7%, 95%CI 20.1–21.3%), the prevalence of self-reported diabetes was greater in White Americans (38.6%, 95%CI 25.9–51.2%) with an incident HCC compared with their counterpart in African Americans (23.2%, 95%CI 17.8–28.6%). The difference in diabetes association with HCC between White and African Americans might be explained by the higher proportion of White Americans with both HCV and diabetes at baseline (14.0%) that developed HCC compared to African Americans (6.8%) with both HCV and diabetes that developed HCC. However, this interaction was not statistically significant in our models. A previous study has found that there is a synergism on HCC risk among diabetes, chronic HBV/HCV infection, and alcohol consumption; presumably accelerating the process of fibrosis and progression to cirrhosis. The odds for HCC in patients with diabetes decreases when the patients do not have HBV, HCV, and alcoholic liver diseases [23].

Our findings with respect to both self-reported hepatitis and diabetes indicate that neither of these risk factors account for the observed higher incidence of HCC among African Americans than White Americans in the SCCS population. Indeed, both illnesses were stronger determinants of risk among White Americans than African Americans, so that the elevated HCC risk among African Americans than White Americans occurs predominantly among those not affected by HCV or diabetes. Determinants of this disparity remain to be quantified.

A number of studies have reported an association between BMI and an increase in HCC risks [24,25,26]. A meta-analysis found that compared to individuals with normal weight (BMI 18.5–24.9 kg m^−2^), those who were overweight (BMI 25–30 kg m^−2^) or obese (≥30 kg m^−2^) had a 17% and 89%, respectively, increased risk of HCC [24]. We found no association between BMI and HCC risk in White or overweight African Americans in adjusted models. Obese African Americans were at a lower risk for HCC than normal weight African Americans. In adjusted models, the interaction between BMI and alcohol was assessed and was not significantly associated with liver cancer incidence in both White and African Americans. Additionally, the interaction between BMI and diabetes was not significantly associated with liver cancer incidence in both White and African Americans. In our stratified analyses by sex among African Americans, the association between BMI and HCC risk was not significant in males but was for females. Differences in results may be due to BMI not capturing the association between obesity and HCC [24]. In one study, visceral fat and not BMI was associated with HCC risk [27]. The Multiethnic Cohort Study also found no association between HCC risk and obesity among African Americans but did find an association among White American males [26]. The study suggested that the previously reported relationship between BMI and the risk of HCC was mediated by visceral fat accumulation which is directly associated with hepatocarcinogenesis rather than BMI [27]. African Americans have been shown to have a lower amount of visceral fat compared to White Americans [28]. Visceral abdominal adiposity was also associated with increased risk for hepatic steatosis and fibrosis in HCV as well as non-alcoholic fatty liver disease [27]. Alternatively, liver disease and cancer can cause a decrease in appetite and weight loss prior to diagnosis. To assesses reverse causality between BMI and liver cancer, analyses were stratified by participants with less than or equal to five years or greater than five years of follow-up time (data not shown). There were no significant differences between BMI and liver cancer by follow-up time and race.

Alcohol [29,30] and cigarette smoking [11,31] are well established risk factors for HCC. In this study, cigarette smoking and alcohol consumption were associated with HCC among African Americans but not White Americans in adjusted models, although the interaction term between smoking and alcohol was not significant for White or African Americans. Cigarette smoking status was similar between White and African Americans at baseline, while alcohol consumption was higher among African Americans compared to White Americans. Alcohol and smoking were significantly associated with liver cancer in univariate models among White Americans but was no longer significant after adjusting for other covariates. The hazard ratios were similar between White and African Americans for smoking and alcohol. It is possible that models were underpowered among White Americans since there were 57 incident liver cancer cases. Smoking cessation and reduction in alcohol consumption could reduce the occurrence of HCC.

In our study, self-reported HCV infection at baseline was associated with a 19-fold increased HCC incidence in White Americans and 8-fold increase in African Americans in adjusted models. The overall self-reported HCV prevalence among White Americans was 5.0% (95%CI 4.7–5.3%) and 3.1% (95%CI 3.0–3.3%) among African Americans. However, among White Americans that developed HCC, 50.9% (95%CI 37.9–63.9%) were infected with HCV at baseline compared to 27.0% (95%CI 21.4–32.7%) among African Americans. Despite the recruitment of both White and African Americans from community health center settings, it is possible that fewer African American SCCS participants were tested for HCV compared to White SCCS participants as HCV was self-reported in our cohort. NHANES data reporting HCV prevalence ratios comparing African Americans to White Americans by state show higher HCV prevalence among African Americans compared to White Americans in each of the 12 states included in the SCCS except for Mississippi [32]. Although the seroprevalence being reported is among the entire population and not stratified by SES. At the time of enrollment, guidelines for HCV testing [33] in the US included a 1-time screening in all adults born between 1945 to 1965 and 80% of the SCCS population would qualify for this screening. The SCCS did not assess HCV testing in this population and therefore we cannot make conclusions without testing and diagnosis information. Additionally, we do not have HCV treatment history available on these participants.

HBV infection was not associated with an increased hazard for liver cancer after adjusting for other covariates in both White and African Americans. HBV self-reported seroprevalence was low in the SCCS and less than 1% of those that reported HBV infection developed liver cancer. There were 117 participants who reported both an HBV and HCV infection in the SCCS cohort and only 4 of these developed liver cancer. An interaction analysis between HBV and HCV was not statistically significant in both White and African Americans. The Center for Disease Control reports that ~15% of liver cancers are attributable to HBV infection in the US and HBV infection disproportionality affects Asian Americans and Pacific Islanders [34].

Our study has several strengths and limitations. The strength of this study includes its prospective design, and the use of a large data set from an ethnically diverse and low socioeconomic status cohort. Ascertainment of HCC was believed to be relatively complete via linkage with cancer registries in each of the 12 recruitment states and with the NDI. The study also considered most known and suspected HCC risk factors. Our results should also be read in the context of certain limitations. There were a small number of HCC cases among White Americans which led to wide confidence intervals and there needs to be caution in the interpretation of some of our findings. Several baseline characteristics were self-reported and subjected our analysis to recall errors. Hepatitis B and C were self-reported at baseline and there was no information on the treatment of these diseases. HCV risk factors such as sexual partners and injection drug use were also not captured within the questionnaire. Our questionnaire did not capture information on liver diseases such as cirrhosis, liver fibrosis, and non-alcoholic fatty liver disease and there could be racial differences in disease severity that could affect progression to HCC.

## 5. Conclusions

In conclusion, we have demonstrated that the prevalence and strength of the association with HCC for several known risk factors differed by race. The major risk factors of HCV, diabetes and obesity, seem weaker (or in the case of obesity, non-existent) among African Americans, suggesting that other factors account for the higher incidence rates of HCC among African Americans. It also is possible that other factors, such as genetic susceptibility, vary between White and African Americans and help explain the risk differentials. Further studies are needed to better understand the associations observed in this study and to identify factors that contribute to the racial differences in HCC risk.

## Figures and Tables

**Table 1:** Baseline characteristics of the study population by race.

Characteristics	White Americans	African Americans
Number at risk	18,678	48,906
Number of hepatocellular carcinoma cases	57	237
Follow-up time (months; median (IQR))	110 (89–129)	126 (100–145)
Age at enrollment (years; median (IQR))	52 (46–59)	50 (44–56)
40–49	41.6	49.8
50–59	33.5	33.7
60–69	18.7	12.6
70–79	6.3	4.1
Sex, %		
Male	34.3	41.3
Female	65.7	58.7
BMI category, %		
Normal	25.8	24.5
Overweight	28.6	28.8
Obese	44.7	45.7
Missing	0.8	1.1
Education, %		
<High School	29.0	33.2
High School	35.4	35.2
>High School	35.6	31.6
Missing	0.0	0.0
Household income, %		
<USD 15,000	56.0	61.8
USD 15,000–49,999	34.3	33.5
>USD 50,000	8.6	3.6
Missing	1.1	1.1
Self-reported hepatitis B virus infection, %		
No	95.7	97.2
Yes	2.0	1.5
Missing	2.4	1.3
Self-reported hepatitis C virus infection, %		
No	92.6	95.6
Yes	5.0	3.1
Missing	2.4	1.3
Diabetes, %		
No	79.0	77.8
Yes	20.7	22.0
Missing	0.3	1.3
Among those with diabetes, use of prescription diabetes medication, %		
No	19.4	13.3
Yes	80.6	86.6
Missing	0.0	0.01
Among those with diabetes, number of years with diabetes (years, median (IQR))	6 (2–12)	7 (2–14)
Insurance coverage, %		
No	43.3	43.2
Yes	56.0	56.3
Missing	0.7	0.6
Use of aspirin, % ^a^		
No	85.3	88.2
Yes	14.3	11.5
Missing	0.4	0.4
Use of over-the-counter pain-relievers, ^a^ %		
No	78.0	84.0
Yes	21.6	15.6
Missing	0.4	0.4
Use of prescription pain-relievers, ^a^ %		
No	93.5	96.6
Yes	6.1	3.1
Missing	0.4	0.3
Cigarette smoking status, %		
Never	30.8	36.4
Former	25.6	19.2
Current	43.4	44.2
Missing	0.2	0.2
Heavy alcohol drinkers, ^b^ %		
No	87.1	78.6
Yes	11.7	20.4
Missing	1.2	1.0
Currently employed, %		
No	66.8	61.9
Yes	32.5	37.5
Missing	0.8	0.7
Laborer, including construction worker, ^c^ %		
No	88.9	87.5
Yes	10.4	11.8
Missing	0.8	0.7
Dry cleaning, ^d^ %		
No	98.7	98.3
Yes	0.6	1.0
Missing	0.8	0.7
Farming, ^d^ %		
No	93.3	94.8
Yes	5.9	4.6
Missing	0.8	0.6
Chemical production or use, ^d^ %		
No	96.0	97.2
Yes	3.2	2.1
Missing	0.8	0.7

Abbreviations: IQR, interquartile range; USD, US dollar; BMI, body mass index; prescription pain relievers include Celebrex, Vioxx, or Bextra. ^a^ In the past year, the participant has taken the medication at least two times per week; ^b^ heavy alcohol drinkers were defined as more than one drink per day on average for females and more than two drinks on average for males; ^c^ laborer, including construction worker: whether they worked as laborers for the longest period of their adult life; ^d^ farming, dry cleaning, chemical production or use: whether they worked in the corresponding field for more than 10 years.

**Table 2:** Univariate and multivariable hazard ratios (HR) and 95% confidence intervals (95%CI) for risk factors associated with hepatocellular carcinoma (HCC) incidence among African Americans.

	No Incident Cancer	Incident HCC	Univariate	Multivariable ^a^
			HR (95%CI)	HR (95%CI)
Age at enrollment ^b^				
40	2644	6	0.19 (0.10–0.38)	0.23 (0.12–0.46)
46	2348	10	0.72 (0.63–0.84)	0.71 (0.61–0.82)
50 (Median)	2203	15	1.00 (Ref.)	1.00 (Ref.)
53	1911	11	1.04 (0.90–1.20)	1.12 (0.97–1.31)
68	422	0	0.86 (0.58–1.28)	1.75 (1.14–2.68)
Sex				
Female	28,624	67	1.00 (Ref.)	1.00 (Ref.)
Male	20,045	170	3.81 (2.87–5.06)	2.05 (1.50–2.80)
BMI category				
Normal	11,888	96	1.00 (Ref.)	1.00 (Ref.)
Overweight	13,994	85	0.72 (0.54–0.96)	0.98 (0.72–1.32)
Obese	22,272	56	0.30 (0.21–0.41)	0.65 (0.45–0.95)
Education				
<High School	16,136	91	1.00 (Ref.)	1.00 (Ref.)
High School	17,128	91	0.92 (0.69–1.23)	1.06 (0.78–1.43)
>High School	15,395	55	0.59 (0.42–0.83)	0.78 (0.55–1.11)
Household income				
<USD 15,000	30,061	177	1.00 (Ref.)	1.00 (Ref.)
USD 15,000–49,999	16,325	56	0.55 (0.40–0.74)	0.84 (0.61–1.17)
>USD 50,000	1746	3	0.27 (0.09–0.85)	0.53 (0.17–1.70)
Hepatitis B				
No	47,335	223	1.00 (Ref.)	1.00 (Ref.)
Yes	714	9	2.78 (1.43–5.42)	1.82 (0.93–3.56)
Hepatitis C				
No	46,578	168	1.00 (Ref.)	1.00 (Ref.)
Yes	1471	64	13.07 (9.79–17.44)	7.73 (5.71–10.47)
Diabetes				
No	37,850	182	1.00 (Ref.)	1.00 (Ref.)
Yes	10,720	55	1.17 (0.87–1.59)	1.48 (1.06–2.06)
Cigarette smoking status				
Never	17,792	28	1.00 (Ref.)	1.00 (Ref.)
Former	9364	35	2.44 (1.48–4.01)	1.45 (0.87–2.42)
Current	21,424	174	5.37 (3.61–8.01)	2.91 (1.87–4.52)
Heavy alcohol drinker ^c^				
No	38,275	146	1.00 (Ref.)	1.00 (Ref.)
Yes	9891	91	2.49 (1.92–3.24)	1.59 (1.19–2.11)
Currently employed				
No	30,084	182	1.00 (Ref.)	1.00 (Ref.)
Yes	18,264	55	0.45 (0.33–0.61)	0.73 (0.53–0.99)

Abbreviations: HR: hazard ratio, BMI: body mass index. ^a^ Multivariable model is adjusted for all other variables in the table; ^b^ self-reported age at baseline was analyzed using restricted cubic splines with 4 knots. We present number of cases with and without HCC at each of those knots. Not all ages are presented in the table; ^c^ heavy alcohol drinkers were defined as more than one drink on average for females and more than two drinks on average for males per day over the past year.

**Table 3:** Univariate and multivariable hazard ratios (HR) and 95% confidence intervals (95%CI) for risk factors associated with hepatocellular carcinoma (HCC) incidence among White Americans.

	No Incident Cancer	Incident HCC	Univariate	Multivariable ^a^
			HR (95%CI)	HR (95%CI)
Age at enrollment ^b^				
42	763	0	0.52 (0.26–1.05)	0.50 (0.24–1.04)
52 (Median)	665	6	1.00 (Ref.)	1.00 (Ref.)
66	313	1	0.64 (0.32–1.30)	1.44 (0.69–3.01)
Sex				
Female	12,248	19	1.00 (Ref.)	1.00 (Ref.)
Male	6373	38	4.24 (2.44–7.35)	2.83 (1.58–5.07)
BMI category				
Normal	4806	21	1.00 (Ref.)	1.00 (Ref.)
Overweight	5336	12	0.51 (0.25–1.03)	0.52 (0.25–1.07)
Obese	8325	24	0.65 (0.36–1.16)	0.79 (0.41–1.53)
Education				
< High School	5395	20	1.00 (Ref.)	1.00 (Ref.)
High School	6593	16	0.66 (0.34–1.27)	0.67 (0.35–1.31)
> High School	6632	21	0.87 (0.47–0)	0.82 (0.42–1.59)
Household income				
<USD 15,000	10,423	40	1.00 (Ref.)	1.00 (Ref.)
USD 15,000–49,999	6393	10	0.39 (0.20–0.79)	0.54 (0.26–1.12)
>USD 50,000	1601	7	1.14 (0.51–2.54)	1.88 (0.74–4.75)
Hepatitis B				
No	17,817	53	1.00 (Ref.)	1.00 (Ref.)
Yes	361	4	3.83 (1.39–10.58)	1.66 (0.58–4.73)
Hepatitis C				
No	17,275	28	1.00 (Ref.)	1.00 (Ref.)
Yes	903	29	22.41 (13.33–37.67)	19.24 (10.58–35.00)
Diabetes				
No	14,714	35	1.00 (Ref.)	1.00 (Ref.)
Yes	3849	22	2.61 (1.53–4.45)	3.55 (1.96–6.43)
Cigarette smoking status				
Never	5739	11	1.00 (Ref.)	1.00 (Ref.)
Former	4770	12	1.37 (0.60–3.10)	0.87 (0.38–2.00)
Current	8069	34	2.35 (1.19–4.65)	1.23 (0.56–2.71)
Heavy alcohol drinker ^c^				
No	16,234	42	1.00 (Ref.)	1.00 (Ref.)
Yes	2164	15	2.53 (1.40–4.56)	1.80 (0.95–3.42)
Currently employed				
No	12,438	38	1.00 (Ref.)	1.00 (Ref.)
Yes	6043	19	0.96 (0.55–1.67)	1.68 (0.90–3.14)

Abbreviations: HR: hazard ratio, BMI: body mass index. ^a^ Multivariable model is adjusted for all other variables in the table; ^b^ self-reported age at baseline was analyzed using restricted cubic splines with 3 knots. We present number of cases with and without HCC at each of those knots. Not all ages are presented in the table; ^c^ heavy alcohol drinkers were defined as more than one drink on average for females and more than two drinks on average for males per day over the past year.

## Data Availability

Restrictions apply to the availability of these data. Data were obtained from the Southern Community Cohort Study and are available at https://www.southerncommunitystudy.org/ (accessed on 11 December 2018) with the permission from the Southern Community Cohort Study.

## Supplementary Materials

The following are available online at https://www.mdpi.com/article/10.3390/cancers13153710/s1, Table S1: Univariate and multivariable hazard ratios (HR) and 95% confidence intervals (95%CI) for risk factors associated with liver cancer incidence among African American females. Table S2: Univariate and multivariable hazard ratios (HR) and 95% confidence intervals (95%CI) for risk factors associated with liver cancer incidence among African American males. Table S3: Baseline characteristics of the study population by race and sex.
