# Supplementary material for: Racial Differences in Hepatocellular Carcinoma Incidence and Risk Factors among a Low Socioeconomic Population
Source: Cancers (Basel). 2021 Jul 23;13(15):3710. doi: 10.3390/cancers13153710 (PMC8345125; doi:10.3390/cancers13153710)
Supplement: Supplementary file 1 [file cancers-13-03710-s001.zip › cancers-1209295-supplementary.pdf]

cancers

MDPI

Article

Racial Differences in Hepatocellular Carcinoma Incidence and Risk Factors among a Low Socioeconomic Population

Sylvie Muhimpundu [1], Rebecca Baqiyyah N. Conway [2,3], Shaneda Warren Andersen [4,5], Loren Lipworth [1], Mark D. Steinwandel [6], William J. Blot [1,6], Xiao-Ou Shu [1] and Staci L. Sudenga [1,*]

[1] Division of Epidemiology, Vanderbilt University Medical Center, Nashville, TN 37232, USA; sylvie.muhimpundu@vanderbilt.edu (S.M.); loren.lipworth@vumc.org (L.L.); william.j.blot@vumc.org (W.J.B.); xiao-ou.shu@vumc.org (X.-O.S.)
[2] School of Community and Rural Health, University of Texas Health Science Center at Tyler, Tyler, TX 75708, USA; rebecca.conway@aaepi.org
[3] American Academy of Epidemiology, Inc., Tyler, TX 75701, USA
[4] Department of Population Health Sciences, School of Medicine and Public Health, University of Wisconsin-Madison, Madison, WI 53706, USA; snandersen@wisc.edu
[5] Cancer Prevention and Control, University of Wisconsin Carbone Cancer Center, Madison, WI 53706, USA
[6] International Epidemiology Institute, Rockville, MD 20850, USA; mark.d.steinwandel@vumc.org
* Correspondence: staci.sudenga@vumc.org

Supplementary

Citation: Muhimpundu, S.; Conway, R.B.N.; Warren Andersen, S.; Lipworth, L.; Steinwandel, M.D.; Blot, W.J.; Shu, X.-O.; Sudenga, S.L. Racial Differences in Hepatocellular Carcinoma Incidence and Risk Factors among a Low Socioeconomic Population. *Cancers* **2021**, *13*, 3710. https://doi.org/10.3390/cancers13153710

Academic Editor(s): Donald D. Anthony

Received: 19 April 2021
Accepted: 20 July 2021
Published: 23 July 2021

Publisher's Note: MDPI stays neutral with regard to jurisdictional claims in published maps and institutional affiliations.

Copyright: © 2021 by the authors. Licensee MDPI, Basel, Switzerland. This article is an open access article distributed under the terms and conditions of the Creative Commons Attribution (CC BY) license (http://creativecommons.org/licenses/by/4.0/).

Table S1. Univariate and multivariable hazard ratios (HR) and 95% confidence intervals (95% CI) for risk factors associated with liver cancer incidence among African American females.

	Univariate	Multivariable [a]
	**HR (95% CI)**	**HR (95% CI)**
Age at enrollment [b]		
40	0.32 (0.09–1.08)	0.28 (0.08–0.96)
47	0.93 (0.77–1.12)	0.83 (0.68–1.00)
50 (Median)	1.00 (Ref.)	1.00 (Ref.)
54	0.92 (0.65–1.31)	1.14 (0.79–1.64)
69	1.90 (1.06–3.38)	4.17 (2.13–8.19)
BMI Category		
Normal	1.00 (Ref.)	1.00 (Ref.)
Overweight	0.55 (0.30–1.01)	0.57 (0.30–1.07)
Obese	0.32 (0.18–0.57)	0.48 (0.26–0.88)
Education		
< High School	1.00 (Ref.)	1.00 (Ref.)
High School	0.93 (0.53–1.65)	1.40 (0.78–2.51)
> High School	0.70 (0.38–1.28)	1.14 (0.59–2.18)
Household income		
<$15,000	1.00 (Ref.)	1.00 (Ref.)
$15,000-$49,999	0.39 (0.21–0.72)	0.59 (0.30–1.14)
>$50,000	0.60 (0.15–2.46)	0.92 (0.20–4.25)
Hepatitis B		
No	1.00 (Ref.)	1.00 (Ref.)
Yes	3.94 (1.24–12.55)	2.27 (0.69–7.42)
Hepatitis C		
No	1.00 (Ref.)	1.00 (Ref.)
Yes	24.77 (14.73–41.63)	21.05 (11.95–37.08)
Diabetes		
No	1.00 (Ref.)	1.00 (Ref.)
Yes	1.36 (0.81–2.30)	1.34 (0.77–2.35)
Cigarette Smoking Status		
Never	1.00 (Ref.)	1.00 (Ref.)
Former	1.60 (0.75–3.42)	1.22 (0.57–2.64)
Current	3.34 (1.89–5.90)	2.52 (1.31–4.86)
Heavy Alcohol Drinker [c]		
No	1.00 (Ref.)	1.00 (Ref.)
Yes	2.48 (1.46–4.22)	2.08 (1.15–3.78)
Currently employed		
No	1.00 (Ref.)	1.00 (Ref.)
Yes	0.48 (0.27–0.84)	0.97 (0.53–1.76)

Abbreviations: HR: Hazard Ratio, BMI: body mass index. [a] Multivariable model is adjusted for all other variables in the table; [b] Self-reported age at baseline was analyzed using restricted cubic splines with 4 knots; [c] Heavy alcohol drinkers were defined as more than one drink on average for females.

Table S2. Univariate and multivariable hazard ratios (HR) and 95% confidence intervals (95% CI) for risk factors associated with liver cancer incidence among African American males.

	Univariate	Multivariable [a]
	**HR (95% CI)**	**HR (95% CI)**
Age at enrollment [b]		
40	0.19 (0.08–0.44)	0.23 (0.10–0.53)
46	0.65 (0.57–0.76)	0.68 (0.59–0.79)
49 (Median)	1.00 (Ref.)	1.00 (Ref.)
52	1.28 (1.05–1.55)	1.27 (1.05–1.55)
66	0.64 (0.36–1.15)	0.93 (0.50–1.74)
BMI Category		
Normal	1.00 (Ref.)	1.00 (Ref.)
Overweight	0.91 (0.65–1.27)	1.12 (0.79–1.58)
Obese	0.51 (0.33–0.78)	0.77 (0.48–1.22)
Education		
< High School	1.00 (Ref.)	1.00 (Ref.)
High School	0.92 (0.66–1.29)	0.98 (0.69–1.39)
> High School	0.62 (0.42–0.93)	0.68 (0.44–1.04)
Household income		
<$15,000	1.00 (Ref.)	1.00 (Ref.)
$15,000-$49,999	0.63 (0.44–0.88)	0.97 (0.67–1.41)
>$50,000	0.13 (0.02–0.90)	0.27 (0.04–1.93)
Hepatitis B		
No	1.00 (Ref.)	1.00 (Ref.)
Yes	2.04 (0.90–4.61)	1.59 (0.70–3.59)
Hepatitis C		
No	1.00 (Ref.)	1.00 (Ref.)
Yes	7.49 (5.29–10.61)	5.48 (3.83–7.85)
Diabetes		
No	1.00 (Ref.)	1.00 (Ref.)
Yes	1.40 (0.97–2.03)	1.54 (1.03–2.31)
Cigarette Smoking Status		
Never	1.00 (Ref.)	1.00 (Ref.)
Former	2.47 (1.21–5.04)	1.60 (0.77–3.33)
Current	4.44 (2.40–8.21)	3.09 (1.64–5.82)
Heavy Alcohol Drinker [c]		
No	1.00 (Ref.)	1.00 (Ref.)
Yes	1.60 (1.18–2.17)	1.45 (1.05–2.00)
Currently employed		
No	1.00 (Ref.)	1.00 (Ref.)
Yes	0.45 (0.31–0.64)	0.66 (0.45–0.97)

Abbreviations: HR: Hazard Ratio, BMI: body mass index. [a] Multivariable model is adjusted for all other variables in the table; [b] Self-reported age at baseline was analyzed using restricted cubic splines with 4 knots; [c] Heavy alcohol drinkers were defined as more than two drinks on average for males.

Table S3. Baseline characteristics of the study population by race and sex.

Characteristics	White (n = 18678)			African Americans (n = 48906)		
	Males	Females	p-Value	Males	Females	p-Value
Number at Risk	6411	12267		20215	28691	
Number of liver cases	38	19	<0.0001	170	67	<0.0001
Age at enrollment (years; median (IQR))	51 (45–59)	52 (46–60)	<0.0001	49 (44–55)	50 (45–57)	<0.0001
BMI category, %			<0.0001			<0.0001
Normal	29.61	23.88		36.3	16.1	
Overweight	33.91	25.87		34.4	24.8	
Obese	36.11	49.19		28.6	57.7	
Missing	0.37	1.06		0.6	1.4	
Self-reported Hepatitis B Virus infection, %			<0.0001			<0.0001
No	94.9	69.1		96.5	97.8	
Yes	2.6	1.6		1.9	1.2	
Missing	2.5	2.3		1.6	1.0	
Self-reported Hepatitis C virus infection, %			<0.0001			<0.0001
No	89.4	94.3		93.5	97.0	
Yes	8.1	3.4		4.9	1.9	
Missing	2.5	2.3		1.6	1.0	
Diabetes, %			<0.0001			<0.0001
No	80.6	78.1		82.0	74.8	
Yes	19.2	21.6		17.8	25.0	
Missing	0.23	0.4		0.2	0.2	
Cigarette smoking status, %			<0.0001			<0.0001
Never	19.9	36.5		21.1	47.2	
Former	28.1	24.3		19.3	19.2	
Current	51.9	38.9		59.4	33.4	
Missing	0.2	0.3		0.1	0.2	
Heavy Alcohol drinkers, [a] %			<0.0001			<0.0001
No	78.7	91.5		67.9	86.1	
Yes	20.4	7.1		31.4	12.7	
Missing	0.9	1.4		0.6	1.3	
Currently employed, %			<0.0001			<0.0001
No	68.4	66.0		63.1	61.1	
Yes	31.2	33.1		36.6	38.1	
Missing	0.42	0.9		0.4	0.9	

Abbreviations: IQR, Interquartile range; CHC, Community health clinic; BMI, Body Mass Index; [a] Heavy alcohol drinkers were defined as more than one drink on average for females and more than two drinks on average for males.
